# Association between diet and gallstones of cholesterol and pigment among patients with cholecystectomy: a case-control study in Korea

**DOI:** 10.1186/s41043-017-0116-y

**Published:** 2017-11-23

**Authors:** Yongsoon Park, Doyeon Kim, Ju Seon Lee, Yu Na Kim, Yoon Kyung Jeong, Kyeong Geun Lee, Dongho Choi

**Affiliations:** 1 Department of Food and Nutrition, Hanyang University, 222 Wangsimni-ro, Seongdong-gu, Seoul, 04763 South Korea; 2 Division of Hepatobiliary and Pancreas Surgery, Department of Surgery, Hanyang University College of Medicine, 222 Wangsimni-ro, Seongdong-gu, Seoul, 04763 South Korea

**Keywords:** Cholesterol gallstone, Pigment gallstone, Cholecystectomy, Diet, Korean

## Abstract

**Background:**

The prevalence of cholesterol gallstones is high in Western populations, while pigment gallstones are common in Asian populations. Dietary factors are suggested to be associated with gallstone risk, but their relationship with gallstone type has not been evaluated. This study investigated the association between diet and risk of cholesterol gallstone or pigment gallstone in a Korean population whose dietary pattern and type of gallstone were changed during the last 30 years.

**Methods:**

Patients with cholesterol (*n* = 40) and pigment (*n* = 59) gallstones were recruited after laparoscopic cholecystectomy and were compared with those of age- and sex-matched controls without gallstones (*n* = 99). Dietary intakes were assessed by trained dietitians using a semi-quantitative food frequency questionnaire. Multinomial logistic regression analysis was performed to calculate odds ratios and 95% confidence intervals to examine the associations between diet and risk for type of gallstones adjusted by potential confounders.

**Results:**

Patients with cholesterol gallstone consumed more lipid, animal lipid, beef, pork, and fried food than those with pigment gallstones and control, while patients with pigment gallstone consumed more carbohydrate and noodles than patients with cholesterol gallstone and control. In multinomial logistic regression analysis using control as reference group, dietary pattern with high consumption of beef, pork, and fried food was associated with risk of cholesterol gallstones, while there was no association between the risk of pigment gallstone and dietary pattern. In addition, control consumed more alcohol than patients with cholesterol and pigment gallstones.

**Conclusions:**

The present study suggested consumption of fat from meat and fried foods increased the risk of cholesterol gallstone, and intake of carbohydrate from noodles increased the risk of pigment gallstone.

**Electronic supplementary material:**

The online version of this article (10.1186/s41043-017-0116-y) contains supplementary material, which is available to authorized users.

## Background

Gallstone disease is one of the most prevalent gastrointestinal disorders [1]. Its incidence has increased around the world, including in Korea, over the past 30 years [2]. Gallstones are mostly classified as cholesterol and pigment gallstones; the prevalence of cholesterol gallstones is higher and the prevalence of pigment gallstones is lower in Western populations compared to Asian populations [3]. Development of cholesterol gallstones is related to saturation of cholesterol in bile, cholesterol crystallization and gallbladder stasis [4]. On the other hand, pigment gallstones develop from the release of β-glucuronidase from bacterial infections; this produces calcium salts of unconjugated bilirubin [5].

It has been shown that age, sex, ethnicity, obesity, and family history of gallstone disease are risk factors for gallstone, and diet is a major modifiable risk factor [1]. Previous studies reported that risk of gallstone was positively associated with intake of meat, energy, fat and saturated fat, but negatively associated with intake of vegetable and fiber in Western and Asian population [6–15]. In particular, high meat intake was associated with risk of gallstone disease [6], since consumption of red meat inhibited bile acid transporters by trimethylamine which induced cholesterol gallstone [16]. In addition, previously studies reported that risk of gallstone was positively intake of margarine, cooking oil, trans fatty acids, and refined sugars, but negatively associated with moderate intake of alcohol and coffee in Western population and Japanese [7, 13, 17–19]. The risk of gallstone was also negatively associated with a healthy dietary pattern in Iranian women [20] and a traditional Mexican diet pattern in Mexican-American men [21]. However, none of the above studies differentiated type of gallstones.

A few animal studies reported that intake of high carbohydrate led to formation of pigment gallstone in prairie dogs [22] and hamsters [23]. However, there has been no human study to compared diet and the risk of pigment gallstones.

Pigment gallstones were predominant in Korean people, but the proportion of cholesterol gallstones has been increased > 50% since the late 1990s [24]. The dietary pattern in Korea has become more westernized with more fat and less fiber during the past a few decades [25]. With the change in dietary pattern, the incidence of cholesterol gallstones has increased and pigment gallstones have decreased in Korea [26]. This finding suggests that Koreans may be a good representative population for analyzing the association between diet and gallstone type. Thus, the purpose of present study was to investigate the hypothesis that westernized diet with more meat rich in fat and less fiber was associated with risk of cholesterol gallstones, while carbohydrate rich diet was associated with risk of pigment gallstones.

## Methods

### Subjects

This study was performed from April 2014 to May 2015 at the general surgery clinic, HYU Hospital, Seoul, Korea with gallstone patients (*n* = 135) who underwent laparoscopic cholecystectomy after diagnosed with gallstones. The presence of gallstones was determined by ultrasonography or computed tomography. Removed gallstones were classified into cholesterol gallstone (*n* = 40), pigment gallstone (*n* = 59), and mixed gallstone (*n* = 36) based on external appearance of the gallstone determined by two independent general surgeons. Patients (cases) were excluded if they had following conditions: underwent open cholecystectomy or biliary drainage procedure; serious comorbidity that required long-term hospitalization; and diagnosed mixed gallstones. Age- and sex-matched controls (*n* = 99) with similar demographic characteristics but without gallstones were recruited from same hospital.

This study protocol was conducted according to the guidelines laid out in the Declaration of Helsinki and was approved by the Institutional Review Board of HYU (HYI-14-001-2). Written informed consent was obtained from all participants.

### Demographic data

Information obtained from participants by trained interviewers included age, sex, family history, medical history, regular exercise, smoking, drinking, and taking supplements. Height and weight were obtained from medical records and body mass index (BMI) was calculated. All women were asked about parity, oral contraceptive use, and hormone replacement therapy.

### Dietary assessment

Dietary intake was assessed by registered dietitian using a semi-quantitative food frequency questionnaire (FFQ) of 63 food items commonly consumed by Koreans Health and Nutrition Examination Survey [27]. Frequency of food intake was classified into 10 categories: one, two, or three times per day; four to six times per week; two to three times per week; once per week; two to three times per month; once per month; six to 11 times per year; never or seldom. Dietary intake was analyzed with CAN-pro 4.0 software (Computer Aided Nutritional Analysis Program for professionals, Korean Nutrition Society, Seoul, Korea).

### Statistical analysis

Data were expressed as mean ± SD and a *P* value < 0.05 was considered statistically significant. All data were analyzed using SPSS version 21.0 (Statistical Package for Social Science, Inc., Chicago, IL, USA). Categorical variables were analyzed using chi-square test, and continuous variables were analyzed using ANOVA after adjusting for potential confounders.

Principal component factor analysis was used to generate dietary patterns based on 25 food groups. Factor scores were rotated using orthogonal (varimax) rotation. To identify the characteristics of the factors, the collection of food groups with factor loadings > 0.4 was used. The factor score for each pattern was calculated by summing intake of food by their factor loadings [28]. After obtained factor scores, multinomial logistic regression analysis was performed to calculate odds ratios (ORs) and 95% confidence intervals (CIs) to examine the associations between dietary patterns and risk for cholesterol and pigment gallstones after adjusting for potential confounders such as energy intake, family history of gallstone disease, and drinking, which were significantly different factors among three groups. In addition, energy intake was added as potential confounders for analyzing association between risk of gallstone and intake of nutrients and foods [29].

## Results

### Baseline characteristics of subjects

Patients with cholesterol and pigment gallstones had significantly higher family history of gallstone disease as compared to controls, while controls consumed more alcohol than patients with cholesterol and pigment gallstones (Table 1). However, there were no significantly differences in age, sex, pregnancy experience, oral contraceptive use, hormone replacement therapy, height, weight, body mass index (BMI), medical history, exercise, smoking, and supplement use among three groups.

**Table 1:** Characteristics of controls and patients with cholesterol and pigment gallstones

	Controls (*n* = 99)	Cholesterol gallstone (*n* = 40)	Pigment gallstone (*n* = 59)	*P* value^d^
Age (year)	49.49 ± 14.79	45.98 ± 14.92	52.00 ± 15.70	0.152
Female, *n* (%)	55 (55.6)	25 (62.5)	33 (55.9)	0.739
Experience of pregnancy, *n* (%)	46 (83.6)	20 (80.0)	27 (84.4)	0.897
Contraceptive use, *n* (%)	20 (36.4)	6 (24.0)	12 (37.5)	0.490
Hormone replacement therapy, *n* (%)	11 (20.0)	5 (20.0)	5 (15.6)	0.866
Height (cm)	163.94 ± 8.57	163.94 ± 8.55	163.81 ± 8.93	0.996
Weight (kg)	63.65 ± 10.98	64.28 ± 9.31	66.13 ± 13.09	0.411
Body mass index (kg/m^2^)	23.58 ± 2.88	23.87 ± 2.64	24.51 ± 3.56	0.181
< 18.5 kg/m^2^, *n* (%)	1 (1.0)	1 (2.5)	1 (1.7)	
18.5–22.9 kg/m^2^, *n* (%)	38 (38.4)	12 (30.0)	18 (30.5)	0.746
23.0–24.9 kg/m^2^, *n* (%)	28 (28.4)	13 (32.5)	14 (23.7)	
≥ 25 kg/m^2^, *n* (%)	32 (32.3)	14 (35.0)	26 (44.1)	
Family history of gallstone disease, *n* (%)	1 (1.0)	5 (12.5)	7 (11.9)	0.007
Medical history, *n* (%)^a^	55 (55.6)	22 (55.0)	42 (71.2)	0.116
Regular exercise, *n* (%)^b^	34 (34.4)	14 (35.0)	18 (30.5)	0.858
Smoking, *n* (%)				0.376
Never smoker	66 (66.7)	24 (60.0)	32 (54.2)	
Ex-smoker	16 (16.2)	11 (27.5)	15 (25.4)	
Current smoker	17 (17.2)	5 (12.5)	12 (20.3)	
Drinking, *n* (%)				0.002
Never drinker	19 (19.2)	8 (20.0)	17 (28.8)	
Ex-drinker	4 (4.0)	7 (17.5)	13 (22.0)	
Current drinker	76 (76.8)	25 (62.5)	29 (49.2)	
Use of supplements, *n* (%)^c^	63 (63.6)	29 (72.5)	37 (62.7)	0.547

Data are mean ± SD or number of subjects (percentage distribution) as appropriate

^a^Medical history such as diabetes mellitus, cardio-cerebrovascular disease, digestive system disease, respiratory disease, urinary disease, and women’s disease

^b^Regular exercise was defined as three times a week for ≥ 30 min

^c^Supplements such as vitamins, minerals, n-3 fatty acids, ginseng, and plant extracts

^d^ *P* value was comparison among three groups by ANOVA for continuous variables or chi-square test for categorical variables

### Nutrient and food intake of participants

Patients with cholesterol gallstone consumed significantly higher fat, animal fat, beef, pork, and fried food than patients with pigment gallstone and controls, while patients with pigment gallstone consumed significantly higher carbohydrate and noodles than patients with cholesterol gallstone and controls (Table 2). Control consumed significantly higher alcohol than patients with cholesterol and pigment gallstones. There was no significantly difference on the intake of other nutrients and food among three groups (Additional file 1).

**Table 2:** Intake of nutrients and foods in controls and patients with cholesterol and pigment gallstones

	Controls (*n* = 99)	Cholesterol gallstone (*n* = 40)	Pigment gallstone (*n* = 59)	*P* value
Energy (KJ)	8934.34 ± 2868.10	9271.01 ± 3179.46	9390.97 ± 3413.54	0.575
Carbohydrates (g)	315.46 ± 96.03a	313.18 ± 96.03^a^	340.49 ± 108.01^b^	0.029
Lipids (g)	56.50 ± 28.63a	65.81 ± 35.43^b^	58.66 ± 31.62^a^	0.038
Plant lipid (g)	26.53 ± 13.13	29.17 ± 14.14	29.51 ± 14.68	0.458
Animal lipid (g)	29.97 ± 18.31a	36.64 ± 24.47^b^	29.16 ± 18.86^a^	0.010
Protein (g)	83.17 ± 33.29	91.19 ± 37.79	86.79 ± 38.34	0.158
Plant protein (g)	40.57 ± 13.30	40.97 ± 12.44	43.91 ± 15.88	0.144
Animal protein (g)	42.60 ± 23.75	50.22 ± 30.07	42.88 ± 25.89	0.052
Noodle (frequency/week)	1.81 ± 2.59^a^	2.41 ± 2.41^a,b^	3.13 ± 3.92^b^	0.046
Beef (frequency/week)	1.98 ± 1.96^a^	3.38 ± 3.16^b^	2.09 ± 2.33^a^	0.001
Pork (frequency/week)	2.14 ± 2.29^a^	3.25 ± 3.23^b^	1.93 ± 2.04^a^	0.012
Fried food (frequency/week)	0.68 ± 1.13^a^	1.88 ± 2.72^b^	0.83 ± 1.63^a^	0.001
Alcohol (frequency/week)	8.25 ± 15.48^a^	4.49 ± 12.94^b^	2.85 ± 7.49^b^	0.004

Data are mean ± SD; values with different letters in the same row are significantly different at *P* < 0.05 by ANCOVA after adjusting for energy intake, family history of gallstone disease, and drinking

The factor loading matrix for four major factors was determined to eigenvalues > 1.4 using Scree plot (Additional file 2). Factor 1 reflected high intake of beef, pork, and fried food; factor 2 reflected high intake of white rice, whole grain, vegetable, and legume; factor 3 reflected high intake of tomato, fruit, and mushroom; and factor 4 reflected high intake of egg, poultry, and seafood (Table 3).

**Table 3:** Rotated-factor loading matrix for the four major patterns

Variable	Factor loading^a^			
	1	2	3	4
Beef	0.859	0.041	− 0.016	− 0.018
Pork	0.849	− 0.027	− 0.024	0.077
Fried food	0.799	−0.079	− 0.038	0.058
White rice	− 0.015	0.791	− 0.079	− 0.125
Whole grain	0.031	0.704	− 0.105	− 0.138
Vegetable	− 0.158	0.623	0.391	0.156
Legume	0.039	0.435	− 0.020	0.405
Tomato	− 0.052	0.042	0.745	0.036
Fruit	− 0.004	− 0.068	0.659	0.066
Mushroom	0.017	− 0.050	0.617	− 0.120
Egg	− 0.022	− 0.060	0.027	0.802
Poultry	0.399	− 0.167	0.015	0.514
Seafood	0.229	0.086	0.309	0.490
Hamburger and pizza	0.181	− 0.007	− 0.029	− 0.041
Coffee and green tea	− 0.037	− 0.088	− 0.023	0.028
Processed meat	0.337	− 0.070	− 0.079	0.400
Bread	0.363	− 0.425	0.112	− 0.009
Cracker	0.043	− 0.074	0.041	0.120
Noodle	0.127	− 0.057	− 0.162	− 0.015
Dairy product	− 0.012	− 0.107	0.035	− 0.029
Rice cake	− 0.037	− 0.090	− 0.108	0.221
Sweet potato	− 0.074	− 0.127	− 0.034	0.033
Seaweed	− 0.007	0.354	0.223	0.209
Alcohol	0.038	0.018	− 0.145	0.033
Carbonated drink	0.019	− 0.100	− 0.027	− 0.065
Eigenvalue^b^	3.668	2.230	2.022	1.467

^a^Factor loading over 0.4

^b^Eigenvalues over 1.4 were extracted

In multinomial regression analysis using controls as reference group, the risk of cholesterol gallstone was associated with factor 1 (beef, pork, and fried food), but the risk of pigment gallstones was no associated with specific dietary patterns (Table 4).

**Table 4:** Association between the risk for cholesterol and pigment gallstones and dietary pattern

Factor number	Controls	Cholesterol gallstone			Pigment gallstone		
	OR	OR	95% CI	*P* value	OR	95% CI	*P* value
Factor 1 (beef, pork, and fried food)	1	1.737	1.11–2.73	0.016	0.965	0.56–1.68	0.900
Factor 2 (white rice, whole grain, vegetable, and legume)	1	0.646	0.38–1.09	0.102	0.958	0.63–1.47	0.844
Factor 3 (tomato, fruit, and mushroom)	1	0.771	0.43–1.39	0.388	1.104	0.73–1.68	0.643
Factor 4 (egg, poultry, and seafood)	1	0.792	0.47–1.35	0.388	0.996	0.66–1.50	0.983

*OR* odds ratio, *CI* confidence interval

*P* value was compared to the controls by multinomial logistic regression after adjusting for energy intake, family history of gallstone disease, and drinking

## Discussion

The present study showed that patients with cholesterol gallstone consumed significantly higher fat, animal fat, beef, pork, and fried food, while patients with pigment gallstone consumed significantly higher carbohydrate and noodles. In addition, risk of cholesterol gallstone was associated with dietary pattern consuming beef, pork, and fried food, while there was no association between risk of pigment gallstone and dietary pattern.

There has been no study that differentiated cholesterol and pigment gallstones in relation to diet. Previous studies reported that the risk of gallstone was positively associated with intake of meat [6], fat, and saturated fat in Europeans [7, 8, 13, 19]. Meat, a major source of fat and saturated fat, was positively associated with risk of gallstones, while unsaturated fatty acid was negatively associated [7]. Type of gallstones was not determined in the above studies; it was assumed mostly cholesterol gallstones since cholesterol gallstone was common in European [6–10, 12–14, 17–19]. The present study consistently showed that consumption of meat from beef and pork, and animal fat was positively associated with the risk of cholesterol gallstone. With intestinal microbiota, dietary L-carnitine and trimethylamine abundant in red meat produce trimethylamine N-oxide which may induce cholesterol gallstone formation due to inhibited bile acid transporters in hepatocytes [16]. In addition, intake of meat rich in saturated fatty acids decreased insulin sensitivity [30] and caused gallbladder disease and gallbladder dysmotility [31]. Insulin resistance has been shown as a risk factor for cholesterol gallstone [32]. Hyperinsulinemia could increase the activity of HMG-CoA reductase, the rate limiting enzyme in hepatic synthesis of cholesterol [33], cholesterol saturation index in the bile [34], thus induce cholesterol gallstone formation. Intake of trans fatty acids from margarine and cooking oil used in frying food has also been suggested to associate with the risk of cholesterol gallstone in American men [17]. Dietary trans fatty acids significantly increase plasma triglyceride levels [35] and impair gallbladder motility from reduced gallbladder sensitivity to cholecystokinin [36]. These findings suggested that hypertriglyceridemia induced by intake of trans fatty acids increased the risk of cholesterol gallstone.

A few studies have investigated dietary pattern and gallstone without differentiating gallstone types. In Iranian women, the risk of gallstones was positively associated with the unhealthy dietary pattern including high intake of red and processed meats, high-fat dairy products, eggs, solid fats, snacks, baked potatoes, and refined grains [20]. The present study also showed that the risk of cholesterol gallstone was positively associated with the dietary pattern including high beef, pork, and fried food. One the other hand, the healthy dietary pattern including high intake of vegetables, fruits, low-fat dairy products, vegetable oil, nuts, whole grains, legumes, fruit juice, and fish was negatively associated with the risk of gallstone [20]. Tseng et al. [21] reported that a traditional Mexican diet with high consumption of corn tortillas, chili peppers, and beans was inversely associated with gallstone in Mexican-American men.

Previous studies showed that intake of vegetables and fiber [9, 10, 12, 13] was negatively associated with risk of gallstone formation in Western populations, suggesting that a diet rich in vegetable fiber has a protective effect against gallstone disease. Marcus et al. [37] reported that intake of insoluble fiber decreased intestinal transit time, reduced the biliary deoxycholic acid, and decreased cholesterol saturation index. However, fiber intake was not associated with risk of cholesterol and pigment gallstone in the present study. This discrepancy could be due to that previous study compared fiber intake of gallstone but the present study differentiated type of gallstone. In addition, fiber intake of Korean (24 g/day) was higher than population of above studies. Risk of gallstone was negatively associated with dietary fiber in American women whose average intake was 7.5 g/day [38], but not associated with dietary fiber in European population whose intake was 22–32 g/day [6], similar to the fiber intake of dietary fiber in the present study.

Pigment gallstones are suggested to be related to poor hygiene and environmental conditions, but not dietary factors [39]. However, few animal studies reported the association between carbohydrate intake and pigment gallstone. A carbohydrate-rich diet increased biliary concentration of phospholipids, calcium, and bilirubin, leading to formation of bilirubinate crystals in prairie dogs [22]. Lee et al. [23] also showed that a high carbohydrate diet increased the incidence of pigment gallstones in Syrian golden hamsters, suggesting that carbohydrates are a relatively weak stimulator of cholecystokinin. On the other hand, previous studies have consistently reported that intake of refined sugars was positively associated with risk of gallstone in Europeans [7, 13, 18]. A diet rich in refined sugar increase insulin, hepatic cholesterol synthesis, and bile cholesterol saturation and impair gallbladder motility, resulting in an increase of gallstone formation [40]. In the present study, intake of carbohydrates was positively associated with risk of pigment gallstone; however, intake of refined sugars was not analyzed due to unavailable data.

Previous studies reported that moderate alcohol consumption was negatively associated with risk of gallstone in European population [18, 41] and the protective effect of alcohol against gallstone regardless of the type of alcohol [42]. Consumption of alcohol has been shown to stimulate cholecystokinin release [43], increased intestine motility [44] and plasma level of HDL-cholesterol [45], leading to prevention of biliary stasis and reduction of bile cholesterol saturation [46]. The present study consistently showed that controls consumed more alcohol than cholesterol and pigment gallstones.

A meta-analysis [47] showed that consumption of coffee, particularly caffeinated coffee was positively associated with risk of gallstone disease in Western population, since intake of coffee could have protective effect by stimulating for the secretion of cholecystokinin, leading to increased gallbladder motility [48]. However, intake of coffee or green tea was not associated with risk of gallstone in Japanese [49]. The present study also shows no association between coffee and tea consumption and risk of gallstone.

This study has a few limitations. First, the sample size was small, which could limit the statistical power to detect differences in the diet. Second, adjustment for potential confounding factors could not eliminate the possibility of residual confounding. Third, in the factor analysis, the number of extracted factors and choice of rotation method was subjective.

## Conclusions

In conclusion, our data suggest that intake of red meat from beef and pork, and animal lipid was positively associated with risk of cholesterol gallstone, while intake of carbohydrate and noodles was positively associated with risk of pigment gallstones, suggesting diet influenced the type of gallstone formation.

## Additional files

Additional file 1: Intake of nutrients and foods in controls and patients with cholesterol and pigment gallstones. (DOC 66 kb)

Additional file 2: Scree plot. (DOC 69 kb)

## Abbreviations

ANCOVA: Analysis of covariance
ANOVA: Analysis of variance
BMI: Body mass index
CAN-pro: Computer aided nutritional analysis program for professionals
CIs: Confidence intervals
FFQ: Food frequency questionnaire
HDL: High-density lipoprotein
HMG-CoA: 3-hydroxy-3-methylglutaryl-coenzyme A
ORs: Odds ratios
SPSS: Statistical package for social science
