# Supplementary material for: Association between diet and gallstones of cholesterol and pigment among patients with cholecystectomy: a case-control study in Korea
Source: J Health Popul Nutr. 2017 Nov 23;36:39. doi: 10.1186/s41043-017-0116-y (PMC5701373; doi:10.1186/s41043-017-0116-y)
Supplement: Supplementary file 1 — Intake of nutrients and foods in controls and patients with cholesterol and pigment gallstones. (DOC 66 kb) [file 41043_2017_116_MOESM1_ESM.doc]

**Table S1.** Intake of nutrients and foods in controls and patients with cholesterol and pigment gallstones

|  | Controls  (n=99) | Cholesterol gallstone  (n=40) | Pigment gallstone  (n=59) | *P*-value |
| --- | --- | --- | --- | --- |
| Energy (KJ) | 8934.34 ± 2868.10 | 9271.01 ± 3179.46 | 9390.97 ± 3413.54 | 0.575 |
| Carbohydrate (g) | 315.46 ± 96.03^a^ | 313.18 ± 96.03^a^ | 340.49 ± 108.01^b^ | 0.029 |
| Fat (g) | 56.50 ± 28.63 | 65.81 ± 35.43 | 58.66 ± 31.62 | 0.038 |
| Plant fat (g) | 26.53 ± 13.13 | 29.17 ± 14.14 | 29.51 ± 14.68 | 0.458 |
| Animal fat (g) | 29.97 ± 18.31^b^ | 36.64 ± 24.47^a^ | 29.16 ± 18.86^b^ | 0.010 |
| Protein (g) | 83.17 ± 33.29 | 91.19 ± 37.79 | 86.79 ± 38.34 | 0.158 |
| Plant protein (g) | 40.57 ± 13.30 | 40.97 ± 12.44 | 43.91 ± 15.88 | 0.144 |
| Animal protein (g) | 42.60 ± 23.75 | 50.22 ± 30.07 | 42.88 ± 25.89 | 0.052 |
| Fiber (g) | 23.68 ± 8.26 | 23.33 ± 7.32 | 26.59 ± 10.81 | 0.121 |
| Vitamin A (μg RE) | 938.40 ± 561.86 | 893.47 ± 497.43 | 1147.91 ± 949.61 | 0.194 |
| Vitamin D (μg) | 3.21 ± 1.93 | 3.74 ± 2.66 | 3.71 ± 2.17 | 0.466 |
| Vitamin E (mg) | 15.98 ± 6.03 | 17.35 ± 6.84 | 17.14 ± 8.21 | 0.780 |
| Vitamin C (mg) | 121.96 ± 60.29 | 116.08 ± 59.28 | 143.25 ± 87.21 | 0.158 |
| Thiamin (mg) | 1.43 ± 0.52 | 1.48 ± 0.58 | 1.52 ± 0.63 | 0.765 |
| Riboflavin (mg) | 1.35 ± 0.55 | 1.42 ± 0.63 | 1.53 ± 0.75 | 0.348 |
| Niacin (mg) | 17.30 ± 6.90 | 18.45 ± 8.08 | 18.18 ± 8.23 | 0.804 |
| Vitamin B_6_ (mg) | 1.78 ± 0.61 | 1.84 ± 0.66 | 1.92 ± 0.78 | 0.828 |
| Folate (μg) | 588.97 ± 198.29 | 558.35 ± 187.93 | 651.61 ± 304.65 | 0.063 |
| Vitamin B_12_ (μg) | 9.60 ± 4.81 | 9.12 ± 4.49 | 10.70 ± 6.19 | 0.230 |
| Calcium (mg) | 578.43 ± 256.90 | 601.75 ± 250.81 | 654.66 ± 282.67 | 0.417 |
| Sodium (g) | 4.40 ± 1.85 | 4.41 ± 1.81 | 4.96 ± 2.42 | 0.135 |
| Potassium (g) | 3.11 ± 1.14 | 3.15 ± 1.21 | 3.48 ± 1.53 | 0.303 |
| Magnesium (mg) | 87.07 ± 40.67 | 90.70 ± 40.78 | 98.50 ± 53.65 | 0.517 |
| Iron (mg) | 16.49 ± 5.51 | 16.66 ± 5.29 | 17.92 ± 6.74 | 0.295 |
| Zinc (mg) | 12.03 ± 4.21 | 13.04 ± 5.00 | 12.81 ± 5.08 | 0.401 |
| Selenium (μg) | 110.17 ± 43.33 | 114.96 ± 45.00 | 112.15 ± 50.95 | 0.518 |
| Cholesterol (mg) | 377.59 ± 198.55 | 421.26 ± 210.86 | 397.73 ± 213.19 | 0.572 |
| White rice (Frequency/wk) | 16.62 ± 8.38 | 13.93 ± 8.49 | 14.71 ± 7.72 | 0.140 |
| Whole grain (Frequency/wk) | 10.28 ± 9.33 | 9.25 ± 8.75 | 9.19 ± 8.84 | 0.693 |
| Noodle (Frequency/wk) | 1.81 ± 2.59^a^ | 2.41 ± 2.41^ab^ | 3.13 ± 3.92^b^ | 0.046 |
| Bread (Frequency/wk) | 1.58 ± 2.13 | 2.79 ± 2.95 | 2.00 ± 2.42 | 0.094 |
| Rice cake (Frequency/wk) | 0.79 ± 1.53 | 0.91 ± 2.30 | 1.18 ± 2.15 | 0.476 |
| Legume (Frequency/wk) | 6.16 ± 5.32 | 7.64 ± 6.64 | 7.71 ± 8.14 | 0.230 |
| Sweet potato (Frequency/wk) | 1.44 ± 2.35 | 1.50 ± 2.17 | 1.34 ± 1.82 | 0.610 |
| Beef (Frequency/wk) | 1.98 ± 1.96^a^ | 3.38 ± 3.16^b^ | 2.09 ± 2.33^a^ | 0.001 |
| Pork (Frequency/wk) | 2.14 ± 2.29^a^ | 3.25 ± 3.23^b^ | 1.93 ± 2.04^a^ | 0.012 |
| Processed meat (Frequency/wk) | 0.92 ± 1.87 | 1.29 ± 3.07 | 1.28 ± 3.12 | 0.558 |
| Poultry (Frequency/wk) | 5.86 ± 8.31 | 7.89 ± 12.26 | 4.02 ± 7.70 | 0.094 |
| Egg (Frequency/wk) | 3.54 ± 3.48 | 3.53 ± 2.68 | 3.59 ± 2.87 | 0.974 |
| Seafood (Frequency/wk) | 6.56 ± 5.00 | 5.61 ± 4.42 | 7.55 ± 8.65 | 0.198 |
| Vegetable (Frequency/wk) | 31.00 ± 18.22 | 26.01 ± 18.75 | 36.66 ± 34.16 | 0.063 |
| Tomato (Frequency/wk) | 3.31 ± 5.20 | 4.77 ± 9.37 | 6.59 ± 10.65 | 0.124 |
| Mushroom (Frequency/wk) | 1.90 ± 4.13 | 2.11 ± 2.41 | 5.69 ± 16.20 | 0.109 |
| Seaweed (Frequency/wk) | 10.40 ± 14.34 | 7.24 ± 10.91 | 11.22 ± 12.71 | 0.310 |
| Fruit (Frequency/wk) | 15.28 ± 13.48 | 13.89 ± 13.75 | 20.46 ± 18.59 | 0.154 |
| Dairy product (Frequency/wk) | 3.10 ± 2.98 | 4.36 ± 5.42 | 4.01 ± 4.41 | 0.238 |
| Fried food (Frequency/wk) | 0.68 ± 1.13^a^ | 1.88 ± 2.72^b^ | 0.83 ± 1.63^a^ | 0.001 |
| Hamburger & Pizza (Frequency/wk) | 1.07 ± 2.97 | 0.89 ± 1.44 | 1.00 ± 1.81 | 0.829 |
| Cracker (Frequency/wk) | 1.82 ± 3.67 | 3.77 ± 8.06 | 2.22 ± 3.54 | 0.174 |
| Coffee & Green tea (Frequency/wk) | 15.43 ± 13.33 | 16.15 ± 17.89 | 14.22 ± 14.72 | 0.775 |
| Carbonated drink (Frequency/wk) | 0.85 ± 1.79 | 1.85 ± 3.08 | 3.60 ± 13.68 | 0.075 |
| Alcohol (Frequency/wk) | 8.25 ± 15.48^b^ | 4.49 ± 12.94^a^ | 2.85 ± 7.49^a^ | 0.004 |

Data are mean ± SD; Values with different letters in the same row are significantly different at *P* < 0.05 by ANCOVA after adjusting for energy intake, family history of gallstone disease and drinking.
