# Supplementary figures and images for: Association between diet and gallstones of cholesterol and pigment among patients with cholecystectomy: a case-control study in Korea
Source: J Health Popul Nutr. 2017 Nov 23;36:39. doi: 10.1186/s41043-017-0116-y (PMC5701373; doi:10.1186/s41043-017-0116-y)

**Figure S1.**
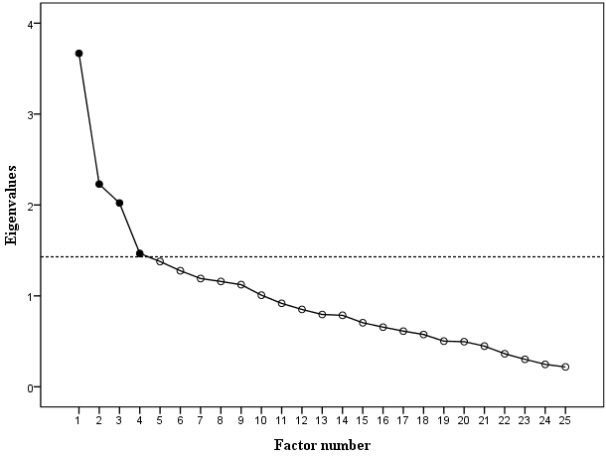
 Scree plot; Eigenvalues over 1.4 were extracted by factor analysis.

Supplement: Supplementary file 2 — Scree plot. (DOC 69 kb) [file 41043_2017_116_MOESM2_ESM.doc]
